# Coptisine ameliorates colitis in mice by modulating cPLA2/TRPM8/CGRP-1 signaling pathways and strengthening intestinal barrier function

**DOI:** 10.1590/1414-431X2025e14349

**Published:** 2025-03-03

**Authors:** Wenbin Wu, Changcheng Shu, Lisheng Chen, Shizhang Wei, Manyi Jing, Hui Li, Haotian Li, Yanling Zhao

**Affiliations:** 1 Health Care Office of the Service Bureau of Agency, Offices Administration of the Central Military Commission, Beijing, China; 2 Graduate School of Chinese PLA General Hospital, Chinese PLA Medical School, Beijing, China; 3 Department of Pharmacy, The Fifth Medical Center, Chinese PLA General Hospital, Beijing, China; 4 National Cancer Center, National Clinical Research Center for Cancer, Cancer Hospital, Chinese Academy of Medical Sciences and Peking Union Medical College, Beijing, China; 5 College of Pharmacy, Chengdu University of Traditional Chinese Medicine, Chengdu, China

**Keywords:** Coptisine, Ulcerative colitis, TRPM8, Network pharmacology

## Abstract

Coptisine (COP), a naturally occurring alkaloid, is recognized for its varied pharmacological impacts and its supportive function in intestinal well-being. However, the role of COP to protect the colonic epithelium in colitis has not been extensively investigated. The objective of this study was to assess the efficacy of COP in ameliorating colitis by investigating intestinal histopathology, mucosal barrier function, and transient receptor potential (TRP) signaling pathways in mice with colon disease compared to a control group, thereby elucidating the underlying mechanisms of its action. The results demonstrated a marked improvement in diarrhea and bleeding, an improvement in general behavioral competencies of the mice, and a decrease in disease activity index (DAI) scores. Histopathological analysis indicated a reduction in intestinal inflammation and an enhancement of intestinal mucosal barrier function. Our research identified that the protein expressions of the TRP family including transient receptor potential cation subfamily M member 8 (TRPM8), transient receptor potential vanilloid 1 (TRPV1), and transient receptor potential ankyrin 1 (TRPA1) were significantly upregulated with COP treatment. Compared with the model, COP markedly downregulated cytosolic phospholipase A2 (cPLA2) levels, while upregulating calcitonin gene-related peptide-1 (CGRP-1) protein expressions. Our study revealed that COP enhanced intestinal barrier function by modulating the cPLA2/TRPM8/CGRP-1 signaling pathway, thus shedding light on the mechanism by which COP mitigates inflammation in the intestinal mucosa. These findings provided new insights on COP as a therapeutic agent in ulcerative colitis (UC).

## Introduction

Ulcerative colitis (UC) is a debilitating inflammatory bowel disease characterized by chronic inflammation of the large intestine and rectum. It presents with a range of symptoms, including abdominal pain, rectal bleeding, and diarrhea, which can significantly impact patients' quality of life and lead to severe complications (1,2). The pathogenesis of UC involves immune dysfunction, microbiome alterations, genetic predisposition, and environmental factors, highlighting the complexity of the disease and the urgent need for effective treatment strategies (3). Research on UC has primarily focused on targeting the immune response and inflammation through biological agents, immunomodulators, and salicylates (4). However, these treatments often face challenges such as symptom recurrence, drug resistance, and adverse effects. Moreover, the underlying mechanisms of the disease remain poorly understood, limiting the development of targeted therapies (5).

Our study aimed to explore the potential of coptisine (COP), a natural alkaloid compound found in *Coptis chinensis* Franch, a traditional Chinese medicinal herb (6). COP has a long history of use in traditional medicine, which may alleviate safety concerns (7). COP has demonstrated pharmacological properties, including antibacterial, anti-inflammatory, and antioxidant effects, which are relevant to the treatment of UC (8). COP could inhibit cell proliferation and induce apoptosis through the phosphatidylinositol 3-kinase-Akt (PI3K-Akt) signaling pathway or block the activation of the nucleotide-binding domain, leucine-rich repeat containing protein 3 (NLRP3) inflammasome by inhibiting caspase-1, which may be related to its anti-inflammatory effects and the reduction of inflammatory diseases (9-12). Importantly, COP can increase the expression of tight junction (TJ) proteins to improve the integrity of the intestinal barrier (11). However, whether there are other pathways related to the intestinal barrier in UC remains largely unaddressed.

**Figure 1:**
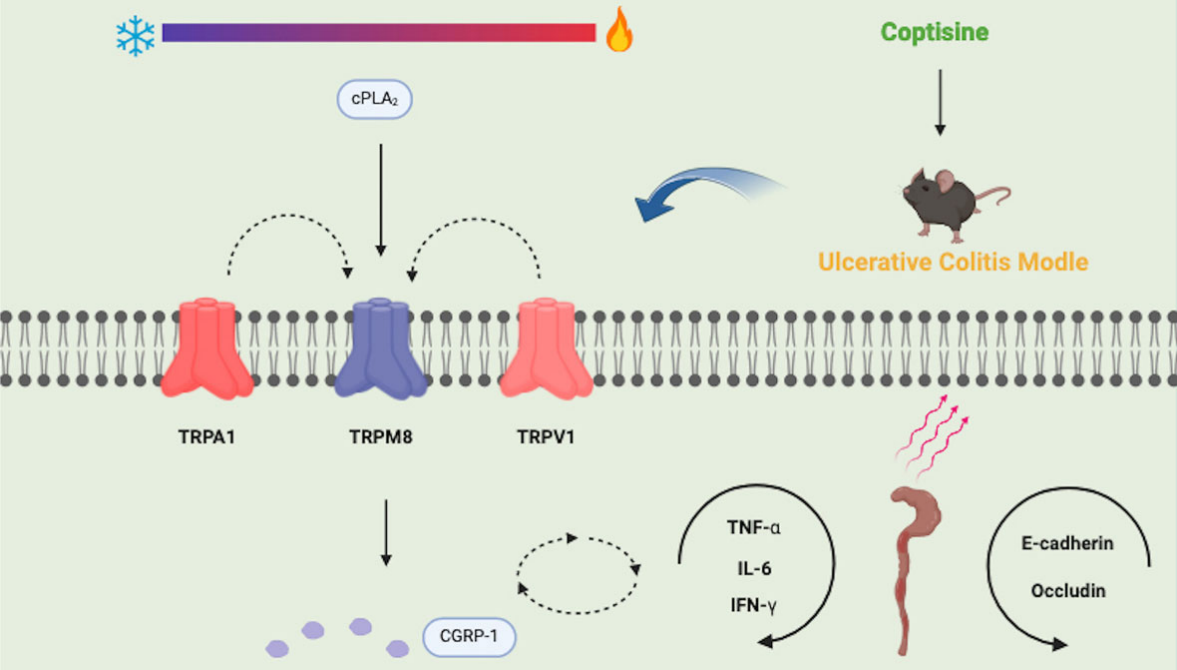
Coptisine has been found to protect against colitis symptoms, improve intestinal barrier function in mice, upregulate TRP family proteins to modulate colon inflammation, reduce intestinal inflammation, and enhance mucosal barrier integrity. Its anti-inflammatory mechanism involves the cPLA2/TRPM8/CGRP-1 signaling pathway, offering a potential therapeutic strategy for treating ulcerative colitis. TNF-α: tumor necrosis factor-alpha; IFN-γ: interferon gamma; IL-6: interleukin 6; TRPM8: transient receptor potential cation subfamily M member 8; TRPV1: transient receptor potential vanilloid 1; TRPA1: transient receptor potential ankyrin 1; cPLA2 cytosolic phospholipase A2; CGRP-1: calcitonin gene-related peptide-1.

Certain members of transient receptor potential (TRP) channel family, predominantly located in colon tissue, play a pivotal role in the pathogenesis of UC (13). Transient receptor potential cation subfamily M member 8 (*TRPM8*) mRNA is highly expressed in colonic dorsal root ganglion neurons, with the corresponding protein diffusely distributed throughout the colon wall's nerve fibers, where it interacts with transient receptor potential vanilloid 1 (TRPV1) channel and transient receptor potential ankyrin 1 (TRPA1) channel to alleviate inflammation in mouse colitis models (14). Patients with UC exhibit elevated levels of cytosolic phospholipase A2 (cPLA2) (15). This enzyme is capable of releasing lipids, including arachidonic acid, which has been demonstrated to inhibit the activation of TRPM8 channel. This inhibition may have implications for the sensory signaling pathways affected in UC (16). TRPM8 activation inhibits the release of tumor necrosis factor (TNF)-α, a pro-inflammatory cytokine, and promotes the secretion of calcitonin gene-related peptide-1 (CGRP-1), which has anti-inflammatory properties. This dual action of TRPM8 channel contributes to the maintenance of intestinal homeostasis and could be a potential target for therapeutic intervention in inflammatory bowel diseases (13,17) (Figure 1). Therefore, our study aimed to investigate the effects of COP on colitis pathologies and the underlying mechanisms by which it strengthens the intestinal barrier function.

## Material and Methods

### Reagents

**Figure 2:**
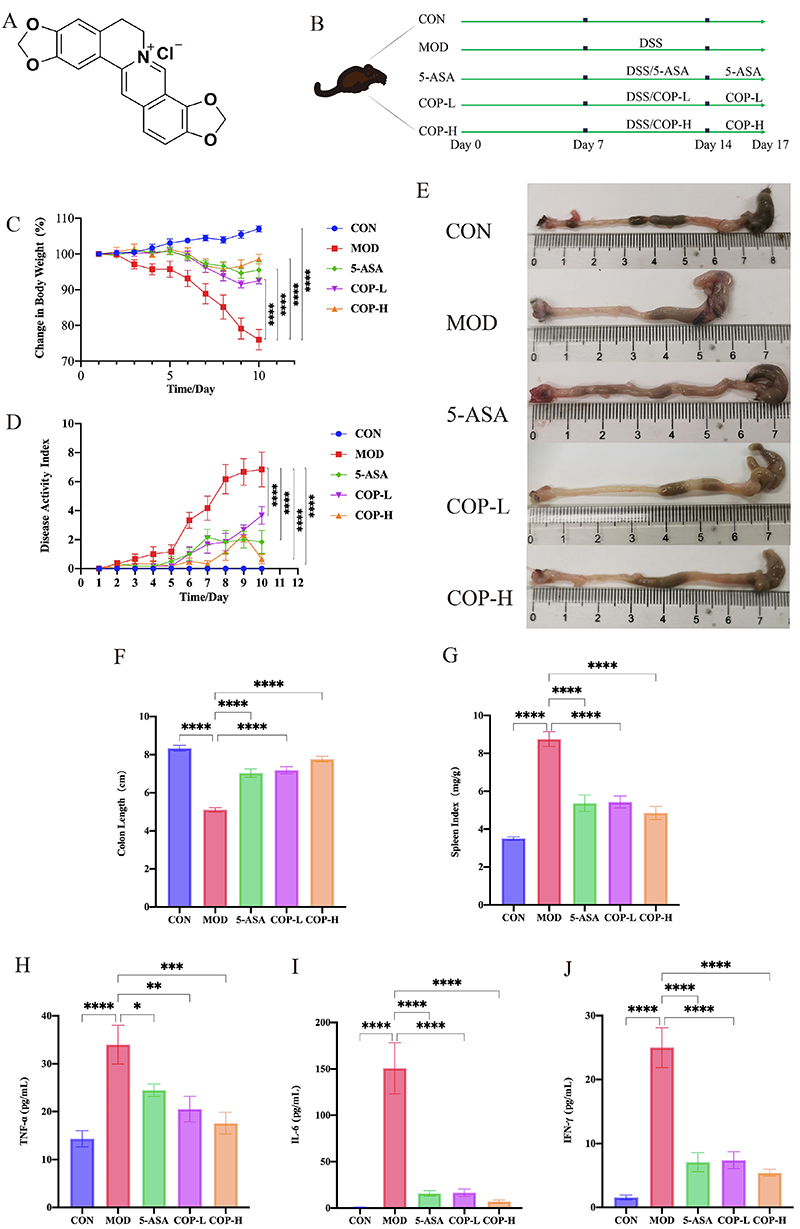
**A**, Molecular formula of coptisine (COP). **B**, Experimental design of COP treatment of dextran sodium sulfate (DSS)-induced ulcerative colitis. **C**, Effect of COP on body weight change. **D**, Effect of COP on disease activity index (DAI) index. **E**, Macroscopic photographs of the colons. **F**, Effect of COP on colon length; **G**, Effect of COP on spleen index. **H**-**J**, Determination of tumor necrosis factor (TNF)‐α, interferon (IFN)-γ, and interleukin (IL)‐6 levels in serum (n=6). Data are reported as means±SE. *P<0.05, **P<0.01, ***P<0.001, ****P<0.0001 *vs* the model (ANOVA). CON: control; MOD: model; 5-ASA: mesalazine; COP-L: low-dose coptisine; COP-H: high-dose coptisine.

COP, with a purity of 99.36%, was acquired from Chengdu Mansite Biotechnology Co., Ltd. (China) (Figure 2A). Mesalazine (5-ASA, 0.25 g) was obtained from Sunflower Pharmaceutical Group Jiamusi Luling Pharmaceutical Co. Ltd. (China). Dextran sodium sulfate (DSS) (36-50 kDa, Catalog Number: 160110) was sourced from MP Biomedicals (Canada). E-cadherin antibody (Catalogue Number: HX14050) and occludin antibody (Catalogue Number: HX20200) were obtained from Beijing Huaxing Bochuang Gene Technology Co., Ltd. (China). The Goat Anti-Rabbit IgG antibody (Catalogue Number: AB0141) was obtained from Abcam Shanghai Trading Co., Ltd. (China).

### Animal studies

C57BL/6 healthy male mice aged 6-8 weeks and weighing 18-22 g were obtained from Sibeifu (Beijing) Biotechnology Co. Ltd. (China) (license number SCXK (Beijing) 2019-0004). The mice were accommodated at the Laboratory Animal Center of the PLA General Hospital Fifth Medical Center, where they were maintained under SPF-grade environmental conditions (relative humidity 55-60%, temperature 20-22°C, 12-h light/dark cycle), with *ad libitum* access to water and food. The Animal Ethics Committee of the PLA General Hospital Fifth Medical Center approved all animal experiments (approval number: IACUC-2021-0022), and the procedures involving animals were performed in strict compliance with standard animal research protocols. The animals were allowed a seven-day acclimatization period prior to the commencement of experiments.

Thirty mice were randomly divided into five groups, with six mice in each group: control (CON) group, model (MOD) group, 5-ASA group, COP low dose (COP-L) group, and COP high dose (COP-H) group. According to the clinical application dose and the dosage conversion between humans and mice, the dose of 5-ASA suspended in saline was set at 200 mg/kg. Based on a two-fold correlation, the doses of COP suspended in saline were set at 80 and 40 mg/kg. From days 8 to 17, the treatment groups received different concentrations of drugs via gavage, while the CON group and the MOD group were administered saline via the same route. From days 8 to 14, the CON group was given purified water, whereas the remaining groups were provided with 3% (weight/volume) DSS solution to drink *ad libitum*. From days 15 to 17, all groups were provided with purified water to drink *ad libitum* (Figure 2B).

### Evaluation of disease activity index (DAI)

The health status of the mice was monitored daily, determining whether they were suffering from UC based on factors such as weight, rectal bleeding, and stool characteristics. During the experiment, the DAI was calculated daily (18). The change in weight was calculated as a percentage of the daily weight relative to the weight at the beginning of the modeling process.

### Sample collections

Upon completion of the experiment, the mice were anesthetized with pentobarbital sodium. After blood collection, cervical dislocation was performed to euthanize the mice humanely. The collected blood was left to clot at 4°C for 1 h before being centrifuged at 1006.2 *g* for 12 min at 4°C to obtain the serum, which was subsequently stored at -80°C for subsequent analysis. Subsequently, the colon was excised from the anus to the ileocecal junction, and its length was recorded. In parallel, the spleens were removed from the mice, and their wet weights were determined. The spleen index was calculated by dividing the wet spleen weight by the body weight of each mouse (19). Following this, a consistent segment was excised from the same area of the distal colon in each mouse. The segment was then preserved in a 4% paraformaldehyde solution for histopathological assessment. After rinsing the colon tissue with a phosphate buffered saline (PBS) solution, the tissue was promptly frozen using liquid nitrogen. The frozen tissue samples were then stored at -80°C for future research and analysis.

### Cytokines quantification

The LEGENDplexTM assay technology (BioLegend, USA) was utilized to quantify cytokines in the serum of colitis mice. A LEGENDplexTM Mouse Inflammation Panel, sourced from BioLegend, was employed for the analysis, with data acquisition conducted on the Cytek NL-CLC 3000 system. Standard curves, produced using a four-parameter logistic fit with known cytokine concentrations provided by the manufacturer, yielded R2 values of approximately 1, and the quality controls included in the kit functioned as anticipated. The mouse TNF-α, interferon (IFN)-γ, and interleukin (IL)-6 LEGENDplexTM kits (BioLegend) were used to measure the levels of these cytokines in the serum, following the manufacturer's protocol.

### Histopathological assessment

The colon tissue was preserved in 4% paraformaldehyde and then embedded in paraffin, after which it was sliced into sections measuring 5 μm in thickness. These sections were then subjected to staining with hematoxylin and eosin (H&E), alcian blue (AB), and periodic acid-Schiff (PAS). A digital slide scanning system (KF-PRO-120, supplied by Ningbo Jiangfeng Biomedical Information Technology Co., Ltd., China) was employed to scan and analyze the stained sections for subsequent histopathological assessment. The extent of damage to the colon specimens was quantified using a detailed scoring system, ensuring objectivity through a double-blind evaluation method. Inflammatory cell infiltration was scored from 0 to 3, with score 0 indicating no infiltration and score 3, severe and extensive infiltration that results in tissue structure loss. Mucosal damage was scored from 0 to 3, with score 0 indicating no damage and score 3, severe damage where there is a loss of both villi and crypts, while the basement membrane remained intact. Crypt damage was scored from 0 to 4, with score 0 indicating no damage and score 4 indicating the complete absence of both the crypt and epithelium.

### Immunofluorescence (IF)

E-cadherin and occludin antibodies, each diluted to a ratio of 1:200, were incubated overnight at a temperature of 4°C. Following this, a dilution of 1:500 of the goat anti-rabbit IgG antibody was added, after which an incubation period of 30 min at room temperature was allowed to elapse. For the purpose of nuclear visualization, 4',6-diamidino-2-phenylindole (DAPI) staining was performed, and the analysis of the samples was conducted utilizing a KFBIO fluorescence scanner (KF-PRO-020).

### Network-based pharmacological inquiry

The structure of COP and its Canonical SMILES were retrieved from PubChem (https://pubchem.ncbi.nlm.nih.gov/), following which they were uploaded to the PharmMapper server (https://www.lilab-ecust.cn/pharmmapper/) as well as Swiss Target Prediction (http://www.swisstargetprediction.ch/) for the purpose of identifying targets associated with COP. Targets associated with UC were sourced from the Human Gene Database (https://www.genecards.org/), version 5.20. The genes that intersected COP and UC were pinpointed through the analysis of both target sets using Jvenn software (https://jvenn.toulouse.inrae.fr/app/index.html), thereby establishing their commonalities. The protein-protein interaction (PPI) network and gene targets were analyzed via the STRING database v12.0 (https://cn.string-db.org/) and visualized in Cytoscape v3.9.0. Kyoto Encyclopedia of Genes and Genomes (KEGG) and Gene Ontology (GO) analyses were performed using the DAVID database, and the findings are presented with an online bioinformatics tool (https://www.bioinformatics.com.cn).

### Western blotting analysis

Proteins from colon tissues were harvested using RIPA lysis buffer, and the protein concentrations were then assessed with a bicinchoninic acid (BCA) protein assay kit. To fractionate the proteins, an 8% sodium dodecyl sulfate-polyacrylamide gel electrophoresis (SDS-PAGE) was run under reducing conditions, after which the proteins were transferred onto polyvinylidene fluoride membranes (PVDF). The membranes were probed with a specific primary antibody at a 1:1000 dilution overnight at 4°C, followed by an hour-long incubation at room temperature with a horseradish peroxidase (HRP)-conjugated secondary antibody, also at a 1:1000 dilution. After a wash with Tris-buffered saline with Tween (TBST), the membranes were visualized using an enhanced chemiluminescence (ECL) system with high sensitivity. The resulting images were analyzed and quantified with ImageJ software, version 1.8.0 (NIH, USA).

### Statistical analysis

All results were analyzed using the Prism version 9.00 software (GraphPad Software, USA). Data were evaluated using one-way ANOVA with Dunnett's multiple comparison test, and an unpaired Student's *t*-test was applied to analyze the significance between two groups. Statistical significance was indicated by a P<0.05. The data are reported as means±SE of three independent experiments.

## Results

### COP reduced DSS-induced colitis in mice

COP significantly alleviated the symptoms of DSS-induced colitis in mice when administered orally. Mice with colitis commonly displayed notable symptoms from the second day onwards, which included weight loss, diarrhea, and rectal bleeding. The treated mice exhibited significant improvements in diarrhea and bleeding symptoms compared to the MOD group, displayed an improvement in general behavioral competencies, and experienced a notable reduction in the overall DAI score (Figure 2D). Furthermore, both the 5-ASA and the COP-L and COP-H groups significantly prevented colon shrinkage in mice, accompanied by reduced spleen indices (Figure 2E-G). Importantly, the COP-H treatment group exhibited a weight gain trajectory akin to the CON group, suggesting a more potent efficacy (Figure 2C). Compared to the MOD group, varying doses of COP treatment significantly reduced the elevated serum levels of TNF-α, IFN-γ, and IL-6, thereby effectively preventing the development of colitis in mice. Consistent with the previously mentioned observations of reduced serum levels of TNF-α, IFN-γ, and IL-6, immunohistochemical analysis further corroborated that COP treatment diminished the levels of these cytokines (Supplementary Figure S1). This indicated that COP could significantly mitigate the inflammation-induced increases in cytokine levels (Figure 2H-J).

### COP protected the colonic epithelium and mitigated injury to the colonic mucosa

**Figure 3:**
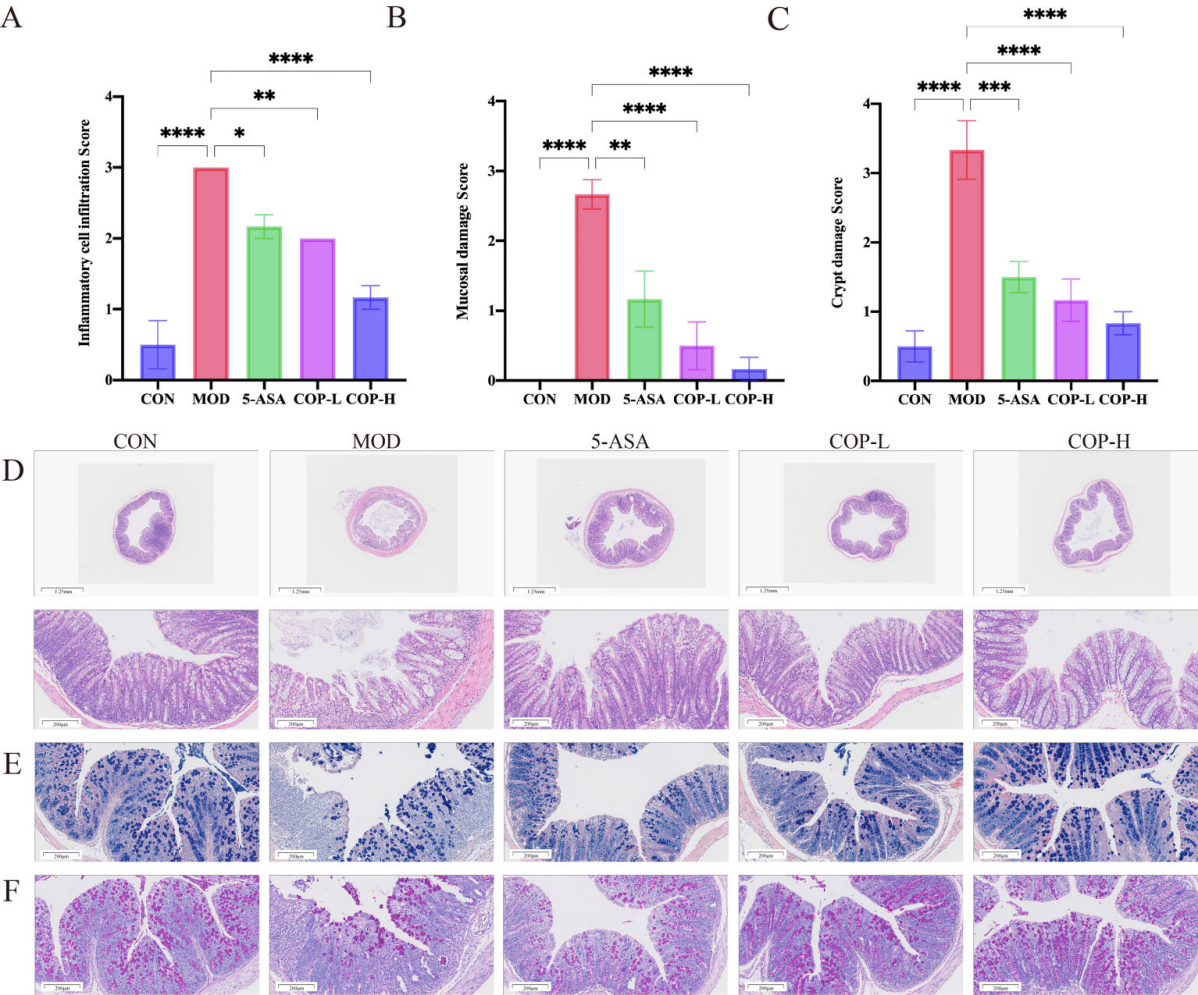
**A-C**, Inflammatory cell infiltration score, mucosal damage score, and crypt damage score in mice with dextran sodium sulfate (DSS)-induced ulcerative colitis. **D**, H&E staining of histopathological sections of mouse colon (scale bar: top 1.25 mm; bottom 200 μm). **E** and **F**, AB and PAS staining of the colon sections of mice with DSS-induced colitis (scale bar: 200 μm). Data are reported as means±SE (n=6). *P<0.05, **P<0.01, ***P<0.001, ****P<0.0001 *vs* the model (ANOVA). CON: control; MOD: model; 5-ASA: Mesalazine; COP-L: low-dose coptisine; COP-H: high-dose coptisine.

Histopathological evaluation provided a detailed analysis of colonic inflammation, revealing that the colonic tissue in the MOD group, compared to the CON group, exhibited pronounced mucosal ulcerations, extensive damage to the colonic mucosal architecture, widespread loss of crypts, a considerable reduction in goblet cells, submucosal hemorrhage, edema, and dense infiltration of inflammatory cells. However, after treatment with COP, the colonic structure in mice became clearly visible. In the COP-L group, there was mild inflammatory cell infiltration and edema in the mucosal and submucosal layers. In the COP-H group, the number of goblet cells and crypt depth in the colon were restored, and inflammatory cell infiltration and mucosal edema were reduced (Figure 3A-D). AB histopathologic staining demonstrated a reduction in goblet cells and mucopolysaccharides in the colons of mice in the MOD group, as opposed to the CON group, after DSS induction. The administration of COP led to an upsurge in goblet cell counts in both the COP-L and COP-H groups, which was accompanied by increased mucus secretion (Figure 3E). Reductions in goblet cell count and in the mucus protein they secrete are frequently linked to the onset of inflammatory conditions. These cells and their secretion are crucial elements of the intestinal mucosal mechanical barrier. We assessed them using PAS staining, which revealed a marked loss of colon mucus protein in the MOD group compared to the CON group, a loss that was notably prevented by COP treatment (Figure 3F).

### COP may prevent colon tissue inflammation by preserving the barrier function of the colon tissue

**Figure 4:**
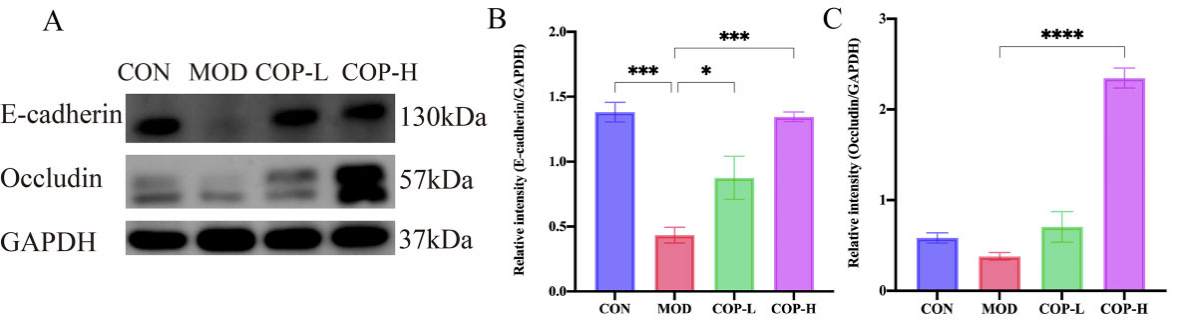
Effect of coptisine (COP) on barrier integrity against dextran sodium sulfate (DSS)-induced colonic mucosal injury. **A**, Western blot analysis for the expression of E-cadherin and occludin in the colon tissue of mice. **B** and **C**, Relative quantification of E-cadherin and occludin by western blot. Data are reported as means±SE, n=3. *P<0.05, ***P<0.001, ****P<0.0001 *vs* the model (ANOVA). The data were normalized prior to statistical analysis. GAPDH: glyceraldehyde-3-phosphate dehydrogenase; CON: control; MOD: model; COP-L: low-dose coptisine; COP-H: high-dose coptisine.

The colonic mucosal barrier's regulatory function is essential for protecting against exogenous antigens and damage. The decreased expression of E-cadherin and occludin in the MOD group compromised the integrity of the colonic mucosa. However, both 5-ASA and various doses of COP were able to maintain the epithelial barrier function, reversing the alterations in E-cadherin and occludin. Notably, the COP-H group demonstrated a marked preservation of the colonic mucosal integrity (Supplementary Figure S2A and B). Western blot results corroborated the findings from IF of increased expression of the tight junction-sealing proteins E-cadherin and occludin (Figure 4A-C). It can be observed that COP treatment significantly enhanced the expression of E-cadherin and occludin in mouse colon epithelial cells, resulting in the formation of a dense fluorescent ring. This observation more vividly demonstrated that COP could reinforce the intercellular connections and had a significant protective effect on intestinal mucosa, which is another mechanism by which COP exerts its therapeutic action in the treatment of colitis.

### COP ameliorated colitis by modulating cPLA2/TRPM8/CGRP-1 signaling pathway

Targets for TRPM8 and TRP channel family, which have been documented to be relevant TRP channels in UC, possibly serve as therapeutic strategies for alleviating intestinal inflammation (17). To verify the effect of TRP targets on colonic epithelium, we performed microscopy and staining. The results illustrated that the expressions of TRPV1, TRPA1, and TRPM8 were reduced in the MOD group. On the contrary, various doses of COP were reversed these alterations. Notably, the COP-H group showed a significant preservation of TRPV1, TRPA1, and TRPM8 targets. CGRP-1, a downstream target of TRPM8, displayed the same trend across different groups. However, cPLA2, as an upstream target of TRPM8, showed the opposite trend (Supplementary Figures S3 and S4).

**Figure 5:**
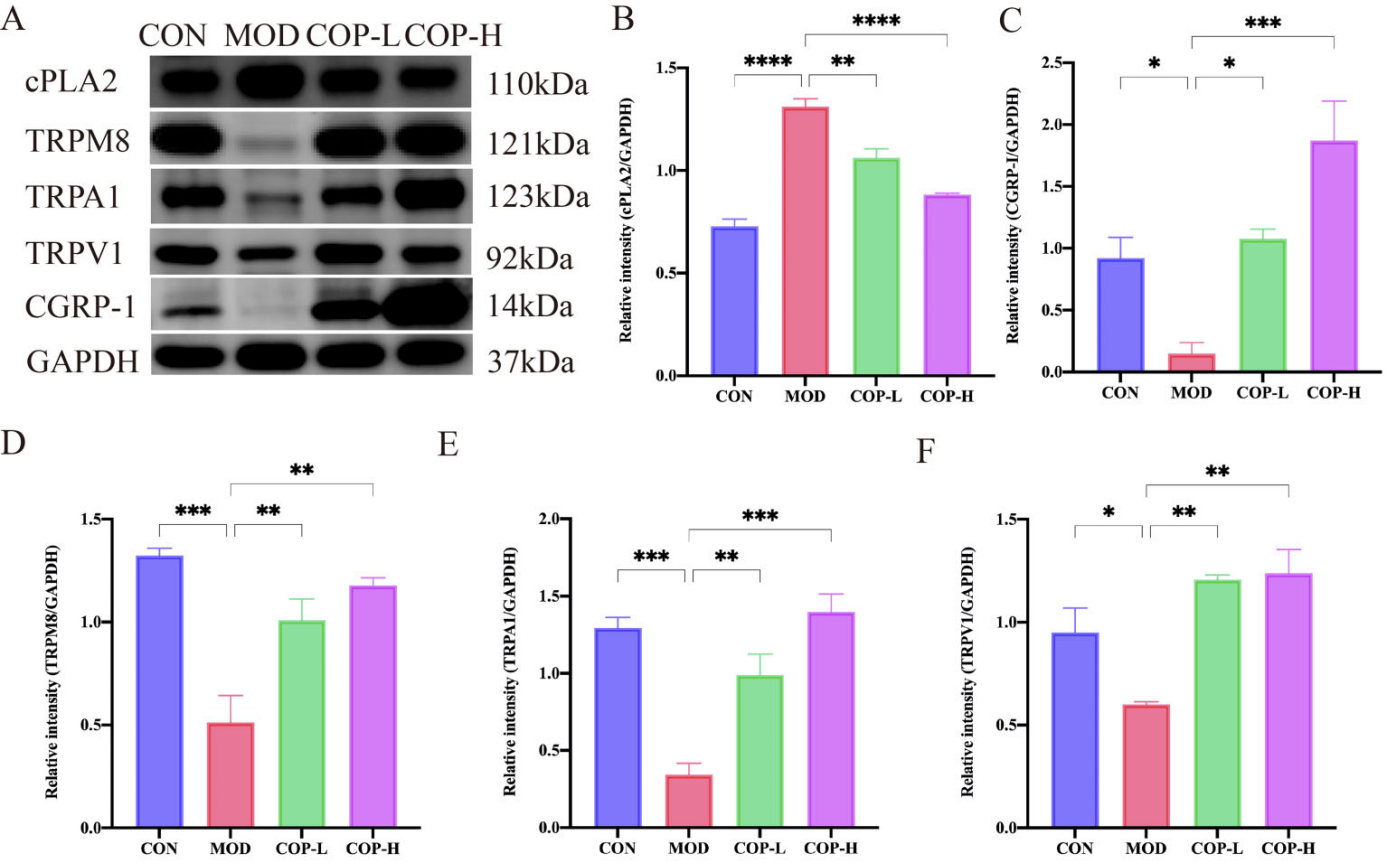
Coptisine (COP) attenuated dextran sodium sulfate (DSS)-induced colitis by regulating TRPM8 signaling pathway in mice. **A**, Western blot analysis for the expression of cPLA2, TRPM8, TRPA1, TRPV1, and CGRP-1 in colon tissue of mice. **B-F**, Relative quantification of cPLA2, CGRP-1, TRPM8, TRPA1, and TRPV1 by western blot. Data are reported as means±SE (n=3). *P<0.05, **P<0.01, ***P<0.001, ****P<0.0001 *vs* the model (ANOVA). The data were normalized prior to statistical analysis. GAPDH: glyceraldehyde-3-phosphate dehydrogenase; CON: control; MOD: model; COP-L: low-dose coptisine; COP-H: high-dose coptisine.

Further experimental studies investigated the regulatory role of COP in the activation of the TRPM8 channel signaling pathway using western blotting analysis (Figure 5A). The data demonstrated a significant upregulation of cPLA2 expression upon DSS administration compared to the control group. This suggested that the upstream signaling target of TRPM8 was inhibited. On the other hand, COP administration significantly suppressed this activation, as indicated by the reduced cPLA2 protein expression. We measured the amounts of TRPV1, TRPA1, and TRPM8 proteins and the evaluations showed that the elevated levels of TRPM8, which interacts with TRPV1 and TRPA1 as a result of DSS, were significantly increased by COP therapy. Moreover, the COP group exhibited an increase in CGRP-1, a downstream target of TRPM8 (Figure 5B-F). All of these results pointed to COP's efficient activation of the TRP pathway, which is exemplified by the TRP channel family.

### Correlation analysis

**Figure 6:**
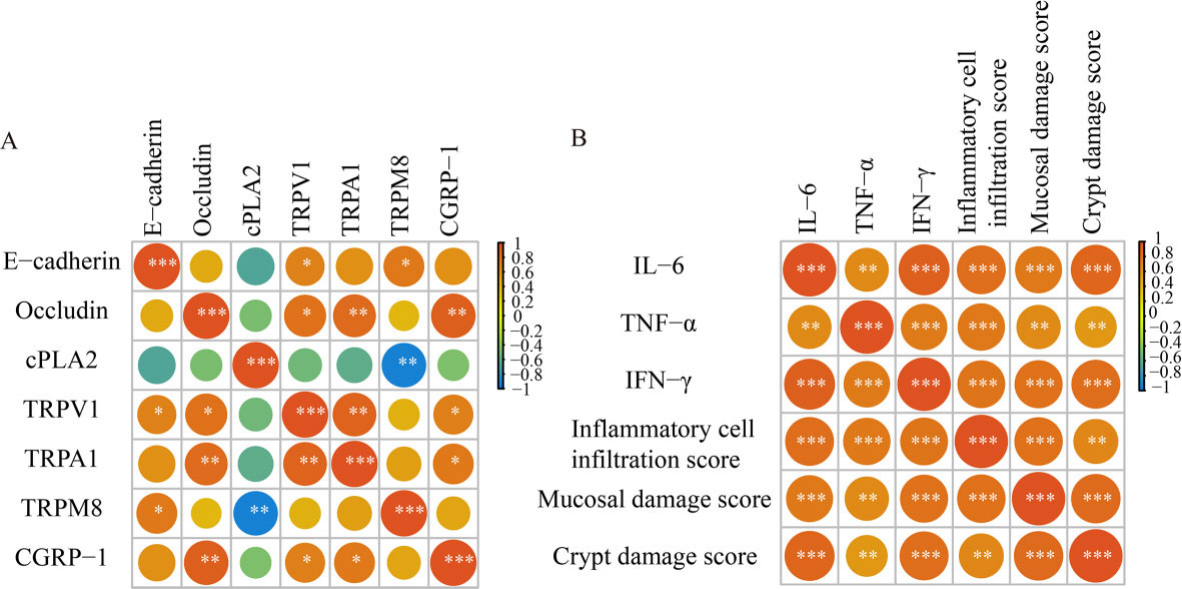
. **A**, Correlation heatmap was constructed for E-cadherin, occludin, and the targets of cPLA2, TRPV1, TRPA1, TRPM8, and CGRP-1. **B**, Correlation heatmap was generated for interleukin (IL)-6, tumor necrosis factor (TNF)-α, interferon (IFN)-γ, and the histopathological scores.

The assessment of the relationships between the targets of TRPM8, among others, and E-cadherin and occludin was performed using the Spearman correlation coefficient analysis, indicating a strong association among them (Figure 6A). Additionally, it was noted that TNF-α, IFN-γ, and IL-6 were significantly associated with the histopathological scores (Figure 6B).

### Elucidating therapeutic targets of COP in ulcerative colitis through network pharmacology and confirming the protective role of TRPM8 signaling pathways in mice with colitis

**Figure 7:**
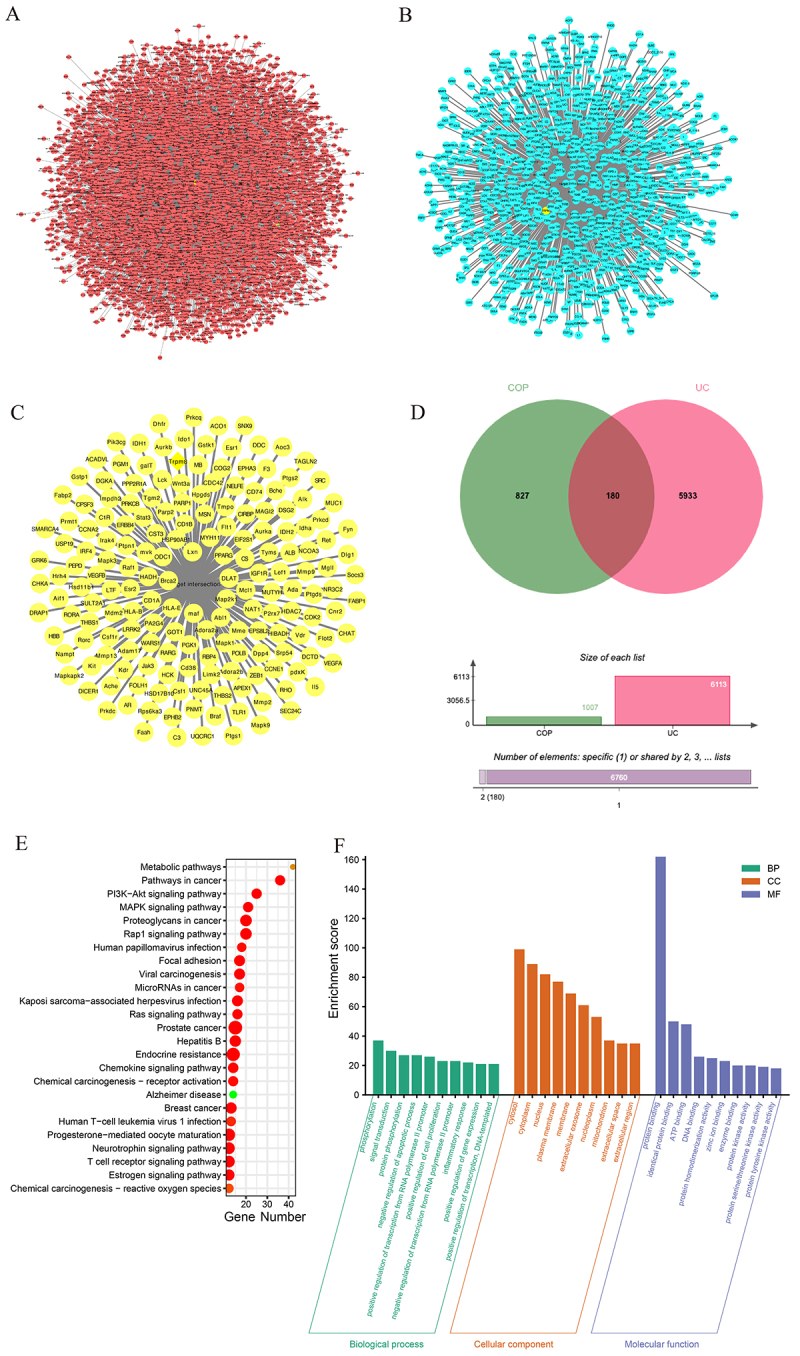
Network pharmacology analysis of coptisine (COP) on ulcerative colitis (UC). **A**, The targets of UC. **B**, The targets of COP. **C**, The potential therapeutic targets of COP for UC. **D**, Venn diagram of potential therapeutic targets of COP for UC. **E**, Kyoto Encyclopedia of Genes and Genomes (KEGG) enrichment analysis. **F**, Gene Ontology (GO) enrichment analysis.

To further validate the therapeutic mechanisms of COP in UC, we examined the genome of UC patients and identified 6,113 genes that are relevant to humans (Figure 7A). Subsequently, we obtained 1,007 potential targets of COP from the PharmMapper and Swiss Target Prediction databases (Figure 7B). Using a Venn diagram, we identified 180 genes that intersected the UC-related genes and the potential targets of COP (Figure 7C and D). Obviously, TRPM8, including its coupling target, was involved. Subsequently, we utilized the DAVID database for GO and KEGG analyses, resulting in the identification of 287 biological process (BP) entries, 57 cellular component (CC) entries, 79 molecular function (MF) entries, and 121 KEGG signaling pathways. After sorting these results by P-value, we extracted the top 25 KEGG signaling pathways and the top 10 GO analysis entries. (Figure 7E and F).

## Discussion

TNF-α, IFN-γ, and IL-6 are pivotal inflammatory mediators. They are commonly up-regulated in inflammatory bowel diseases such as UC, which then stimulates the inflammatory response of the intestinal mucosa, including the activation of immune cells and the breakdown of the intestinal barrier (18,20). TNF-α, a potent inflammatory mediator, can induce the inflammatory response of the intestinal mucosa. It can induce apoptosis of intestinal epithelial cells and increase the permeability of the intestinal mucosa. IFN-γ, predominantly produced by activated T cells and natural killer cells, plays a crucial role in the immune response. It can enhance the activity of macrophages and promote the inflammatory response. IL-6 is a multifunctional cytokine involved in the regulation of immune response and inflammatory processes. Additionally, IL-6 can promote the development of inflammatory bowel disease through the STAT3 signaling pathway (21). Wu et al. (22) suggested that STAT3 can modulate the expression of pain-related genes, including TRP channels expressed in sensory neurons. The activation of STAT3 in immune cells can lead to the production of inflammatory cytokines, which can interact with sensory neurons and augment pain signal transmission through TRP channels (23).

E-cadherin and occludin are pivotal proteins that uphold the integrity of tight junctions between intestinal epithelial cells, thus ensuring the cohesion of the intestinal barrier. Upon exposure to inflammatory triggers such as TNF-α, IFN-γ, and IL-6, these factors can either directly or indirectly influence the expression and function of tight junction proteins. Inflammatory agents may elevate the permeability of the intestinal epithelium and exacerbate inflammation by diminishing the expression of E-cadherin and occludin (24,25). Moreover, the interaction between inflammatory factors and tight junction proteins is reciprocal. For instance, TNF-α can diminish the expression of tight junction proteins by engaging specific signaling cascades (like NF-κB) (26). Conversely, the breakdown of tight junctions may precipitate an increase in the local concentration of inflammatory factors, which can further exacerbate intestinal mucosal inflammation, establishing a self-perpetuating cycle (27). COP might enhance tight junctions by modulating these molecules and augmenting the expression and functionality of E-cadherin and occludin, thereby aiding in the restoration of intestinal barrier function and limiting the infiltration of inflammatory agents. This, in turn, would reduce intestinal permeability and mitigate inflammation.

TRPM8, a key temperature and nociceptive receptor in sensory neurons, possesses the capacity to alleviate colitis (28). Previous research has demonstrated that TRPM8 suppresses the development of colitis induced by trinitrobenzene sulfonic acid (TNBS) or DSS in mice (29). TRPM8 expression has been observed within intestinal cells, and this expression has been found to be associated with the intestinal barrier function in UC (28,30). TRP channels are involved in the inflammatory process of UC (13). These channels can be activated by a variety of endogenous and exogenous stimuli, leading to the influx of calcium ions, which in turn activates downstream signaling pathways and promotes the release of inflammatory mediators such as prostaglandins and cytokines, which play a key role in the inflammatory response of UC (17,31). During inflammation, cytokines and molecules can alter TRP channels, which play roles in intestinal sensation, pain, and barrier integrity, affecting the disease's progression (13).

The results showed that COP effectively reduced DSS-induced impairment in colonic barrier function and inflammatory disorders by targeting TRPM8 activation. DSS administration induced UC-like symptoms and inhibited TRPM8 in the colon, which were attenuated by COP treatment through the same pathway. Phospholipase A2 (PLA2) levels are elevated in patients with UC. Empirical evidence suggested a coordinated involvement of cPLA2 and COX-2 in the immune and inflammatory cascades. The blockade of the cPLA2-COX-2 signaling axis elicits a shift towards M1 macrophage polarization, a process that subsequently modulates the production of reactive oxygen species and contributes to the defense against leishmania. Acting as a pivotal upstream mediator of inflammation, interleukin facilitates the activation of cPLA2, which in turn influences the functional activity of COX-2 and the synthesis of prostaglandin E2. Additionally, cPLA2 has been observed to exert a regulatory inhibition on TRPM8 activation. Activation of cPLA2 results in a decrease of the activation threshold for TRPM8, indicative of a suppressive effect on its activity (13,16). *TRPM8* mRNA is abundant in colonic dorsal root ganglion neurons, with the corresponding protein found throughout nerve fibers of the colon wall. In animal models of post-inflammatory colonic allergy, elevated TRPM8 expression markedly reduced hypersensitivity to mechanical stimulation and alleviated allergy symptoms. Inflammation in mouse colitis models can be alleviated by the activation of TRPM8. Selective activation of TRPM8 reduces the release of inflammatory neuropeptides, inhibits the release of pro-inflammatory cytokines and chemokines, and prevents the accumulation of leukocytes in the colon (14,32).

In addition to lowering inflammatory cytokine levels, TRPM8 activation also lessens the inflammatory response (33). By coupling with TRPV1 and TRPA1, TRPM8 blocks the chemosensory and mechanosensory effects that follow. Researchers suggested a protective role of TRPV1 and TRPA1 in the GI tract (34). Furthermore, co-activation of TRPV1 and TRPM8 or TRPA1 produces an analgesic effect (35). Additional research has indicated that by reducing the production of TNF-α, IFN-γ, and IL-6 and promoting the synthesis of CGRP-1, TRPM8 functions as a protective factor in the intestine (17,29). Additionally, our studies suggested a direct correlation between COP's colon-protective effects and the expression of the TRP channels family proteins. Overall, these findings reveal a previously unexplored mechanism by which COP diminishes inflammation in colitis, specifically through the TRPM8 signaling pathway.

Our findings underscored the potential of TRP channels as therapeutic targets for UC. Depending on the direction of their dysregulation, TRP channels could be targeted with antagonists to reduce cold sensitivity or with agonists to restore normal gut sensation. The combination of TRP channel modulators with existing UC treatments, such as anti-inflammatory drugs or biologics, could potentially enhance treatment outcomes through synergistic effects. Furthermore, the consistent expression patterns of TRP channels observed in UC patients suggest their potential use as diagnostic, prognostic, or therapeutic response biomarkers, which could lead to the development of non-invasive diagnostic tools for UC.

Moreover, the results of network pharmacology have validated existing literature and our experimental findings, thereby confirming the TRPM8 signaling pathway and inflammatory factors such as TNF-α, IFN-γ, and IL-6 as pivotal components in the regulatory role exerted by COP on DSS-induced colitis. The pathway enrichment analysis revealed that several pathways are associated with TRP channels, with the PI3K-Akt signaling pathway and the mitogen-activated protein kinase (MAPK) pathway being the most prominent. Research has shown that the activation of the PI3K-Akt pathway can influence the expression and function of TRP channels (36). MAPK is involved in various biological processes, including cell growth, differentiation, and apoptosis. Specifically, TRPV1 and TRPM8 can be regulated by the MAPK pathway (37).

Additionally, protein kinase activity is intricately associated with protein phosphorylation and tyrosine phosphorylation within the signaling pathway, and the TRP pathway is a crucial mechanism for regulating intracellular calcium levels, osmotic pressure, and other physiological states through these mechanisms (38). By investigating these interactions, we can more effectively elucidate the role of COP in the regulation of TRP channels in colitis, thereby presenting new possibilities for the development of novel therapeutic interventions.

## Conclusions

COP ameliorated colitis in mice by regulating the cPLA2/TRPM8/CGRP-1 pathway, strengthening intestinal barrier function, and modulating inflammatory mediators, which was supported by network pharmacology analysis, offering novel insights into its protecting mechanism. This study is beneficial to a deeper understanding of the naturally occurring alkaloid of COP, and also provides a scientific basis for further regulatory mechanisms of COP on UC and drug development.

## Supplementary Material

Click here to view [pdf].
