# Supplementary material for: Coptisine ameliorates colitis in mice by modulating cPLA2/TRPM8/CGRP-1 signaling pathways and strengthening intestinal barrier function
Source: Braz J Med Biol Res. 2025 Mar 3;58:e14349. doi: 10.1590/1414-431X2025e14349 (PMC11884773; doi:10.1590/1414-431X2025e14349)

Figure S1. Immunohistochemistry results (scale bar: 50 μm). TNF-α: tumor necrosis factor-alpha; IFN-γ: interferon gamma; IL-6: interleukin 6; CON: control; MOD: model; 5-ASA: mesalazine; COP-L: low-dose coptisine; COP-H: high-dose coptisine. *P<0.05, **P<0.01, ***P<0.001 (ANOVA).

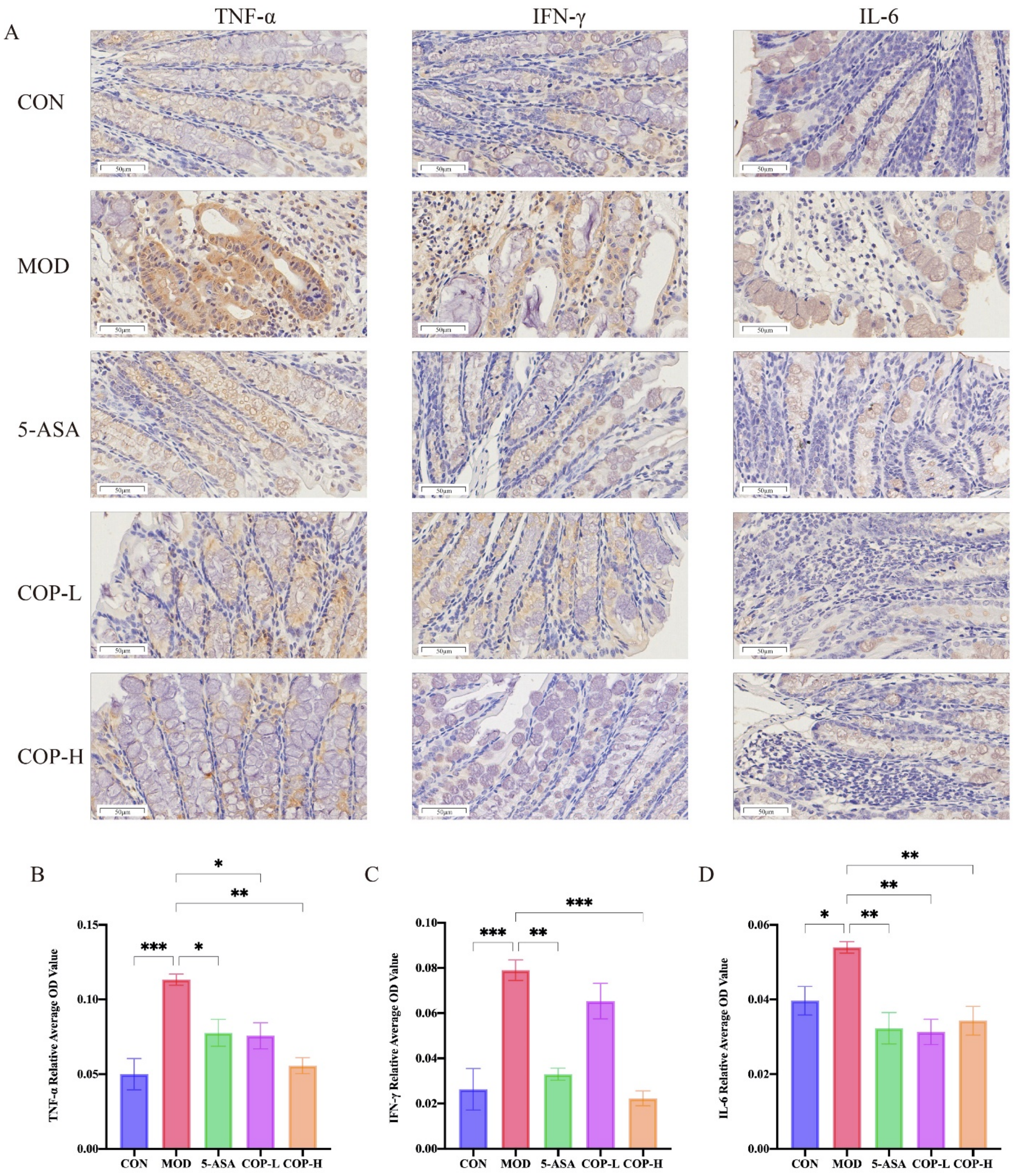

Figure S2. Evaluation of the expression of E-cadherin (**A**) and occludin (**B**) using immunofluorescence microscopy, with both proteins visualized in green. Furthermore, cell nuclei were stained with DAPI, which appear in blue, to assist in the identification and localization of cellular structures (scale bar: 50 μm). CON: control; MOD: model; COP-L: low-dose coptisine; COP-H: high-dose coptisine.

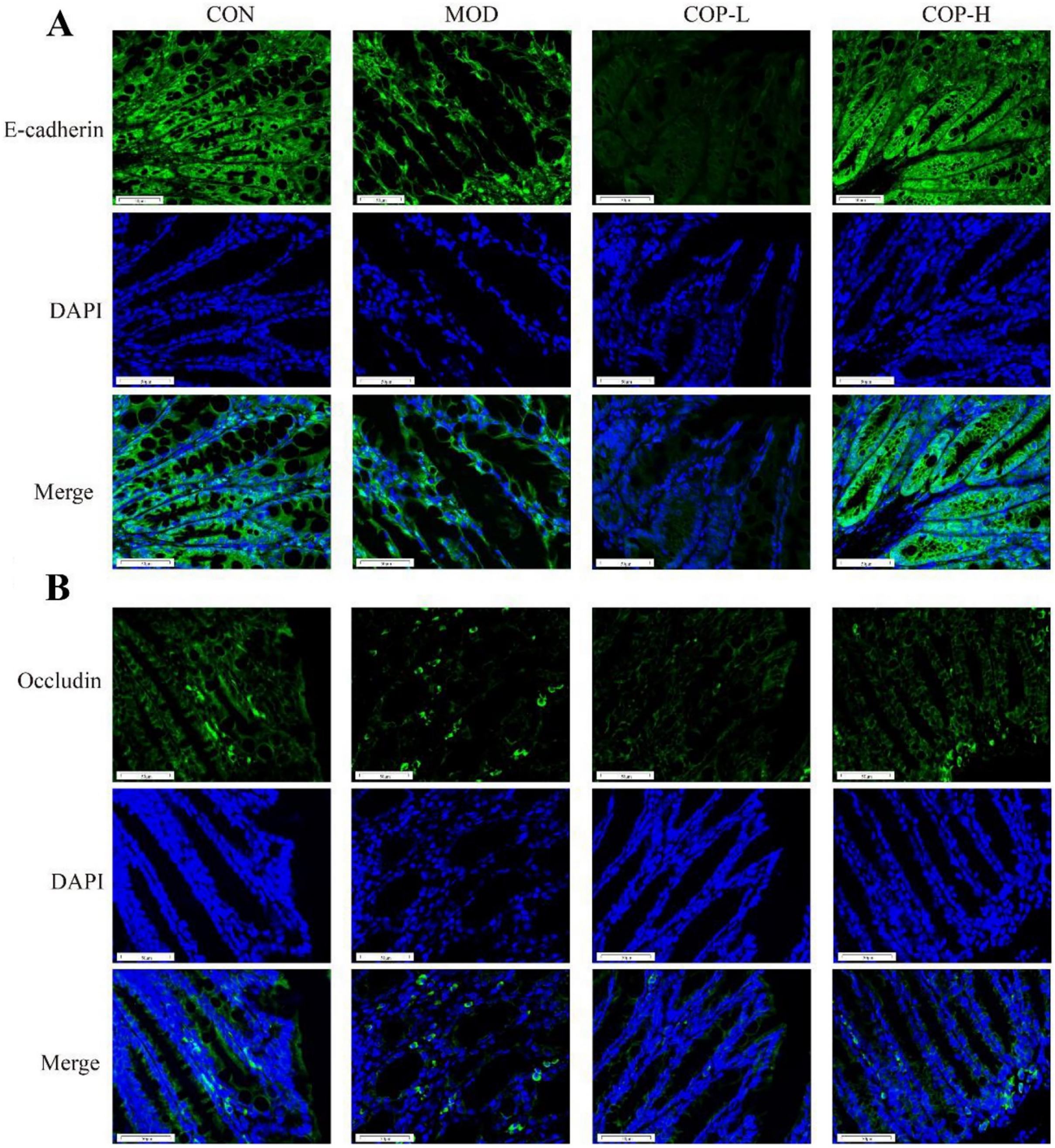

Figure S3. Evaluation of the expression of cPLA2 (**A**), TRPM8 (**B**), and CGRP-1 (**C**) using immunofluorescence microscopy, with all proteins visualized in green. Additionally, cell nuclei were stained with DAPI, which appear in blue, to aid in the identification and localization of cellular structures (scale bar: 50 μm). CON: control; MOD: model; COP-L: low-dose coptisine; COP-H: high-dose coptisine.

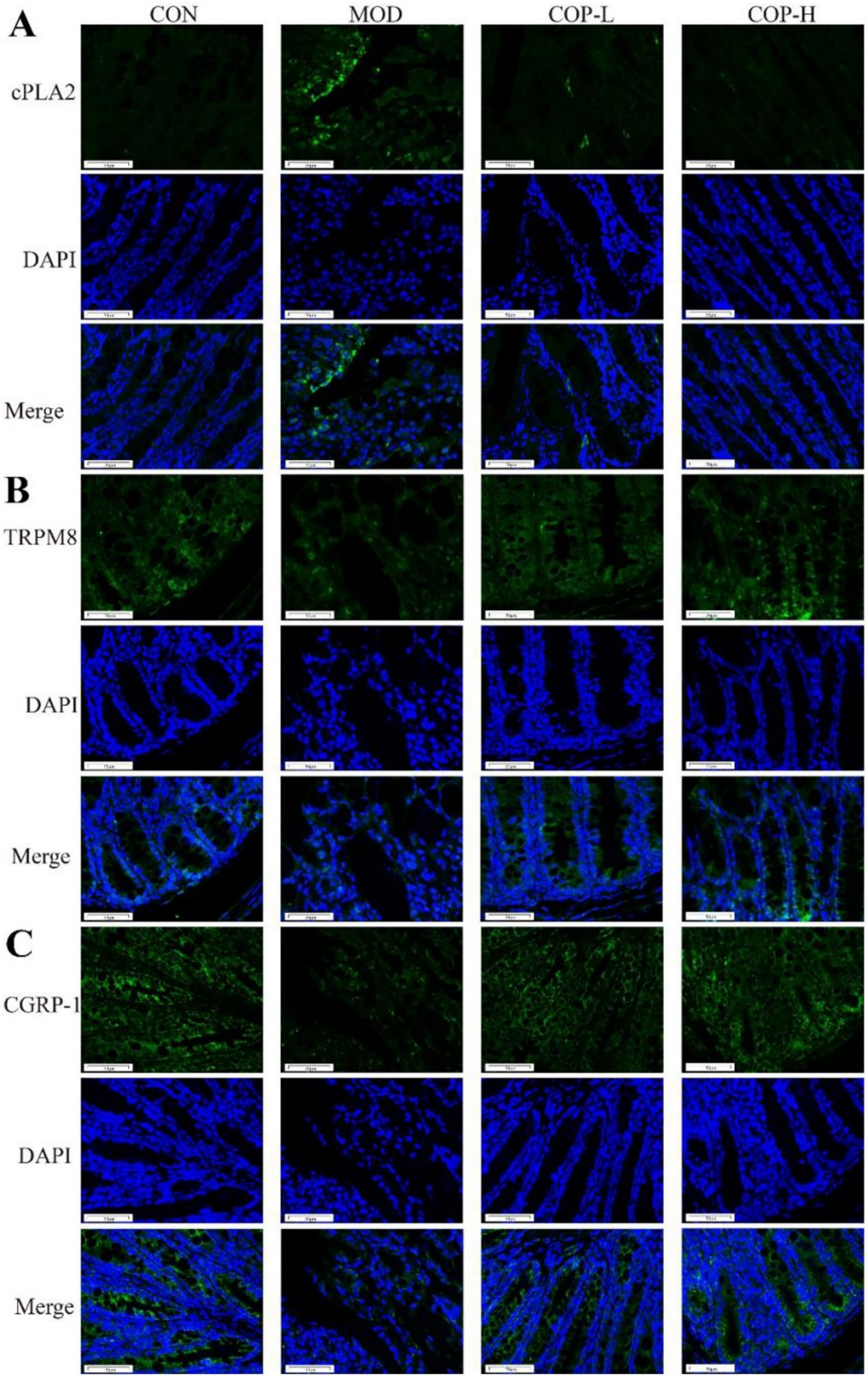

Figure S4. Evaluation of the expression of TRPA1 (**A**) and TRPV1 (**B**) using immunofluorescence microscopy, with all proteins visualized in green. Additionally, cell nuclei were stained with DAPI, which appear in blue, to aid in the identification and localization of cellular structures (scale bar 50 μm). CON: control; MOD: model; COP-L: low-dose coptisine; COP-H: high-dose coptisine.

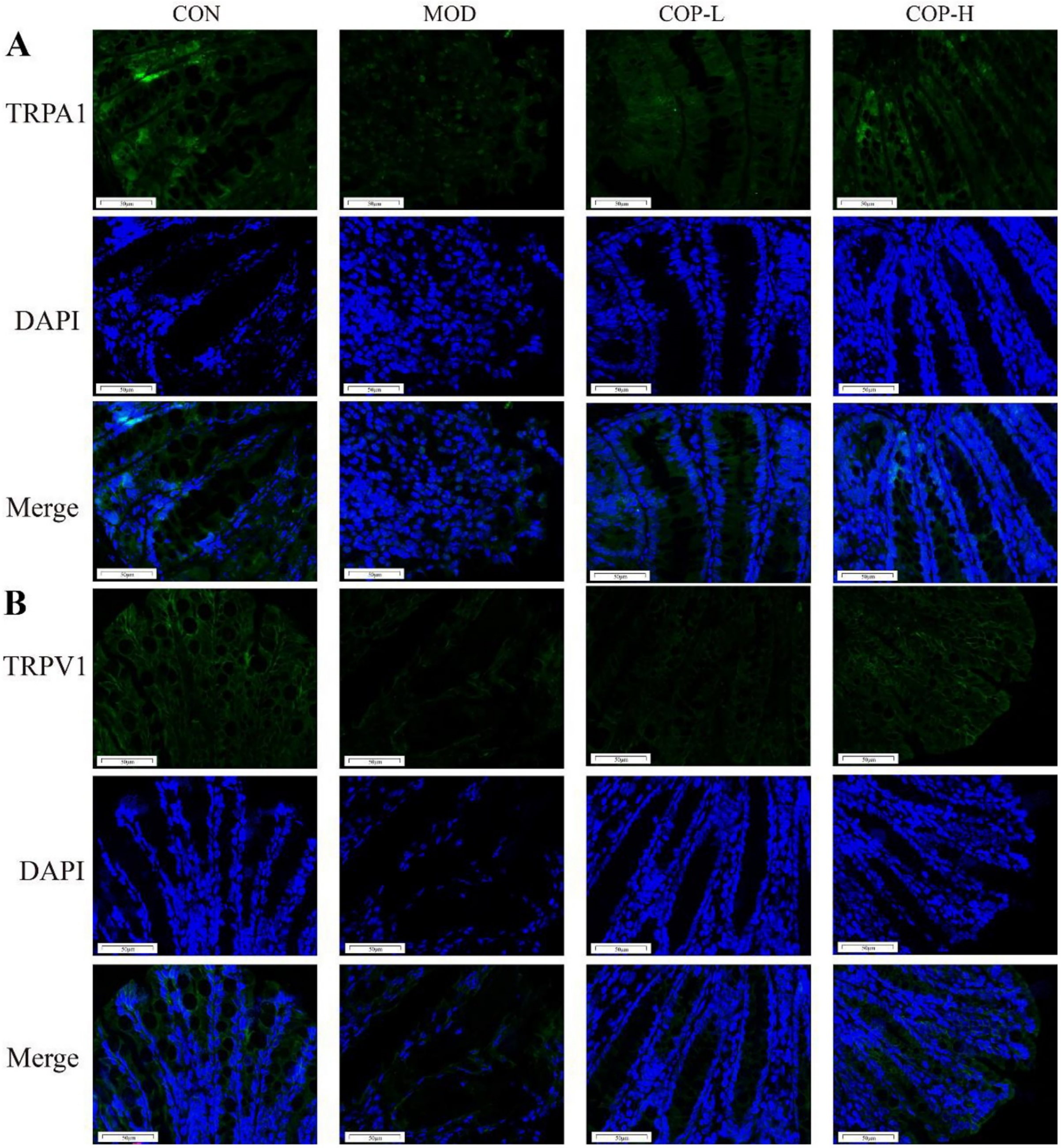

Supplement: Supplementary file 1 [file 1414-431X-bjmbr-58-e14349-suppl.pdf]
